# Atrial Myxoma Presenting as an Atypical Stroke in a Young Patient With a Recent COVID-19 Infection

**DOI:** 10.7759/cureus.26407

**Published:** 2022-06-28

**Authors:** Drew E Moss, Dilpat Kumar, Aditya Mehta, Prashant P Patel

**Affiliations:** 1 Department of Internal Medicine, Western Michigan University Homer Stryker M.D. School of Medicine, Kalamazoo, USA

**Keywords:** embolism, covid, echocardiography, cardiac tumor, stroke, atrial myxoma

## Abstract

Atrial myxomas are the most common primary tumor of the heart and can occasionally present as an ischemic stroke with neurologic symptoms secondary to embolic phenomena. We present a case of a 42-year-old male with multiple cardiovascular risk factors and coronavirus disease 2019 (COVID-19) infection two months prior who presented to the emergency department with unilateral left-sided weakness and paresthesia. After being diagnosed with multifocal ischemic strokes, further evaluation utilizing a transesophageal echocardiogram (TEE) revealed a 5 × 2 cm left atrial myxoma prolapsing the mitral valve, which was the presumed cause of the patient’s strokes. The myxoma was successfully removed via robotic thoracoscopy. Our case demonstrates the importance of considering atrial myxoma in the evaluation of stroke in young and middle-aged patients even in the presence of multiple cardiovascular and thrombotic risk factors.

## Introduction

Atrial myxomas are the most common primary tumor of the heart and are most often found in the left atrium [1]. Although a minority of patients with atrial myxomas may be asymptomatic, the majority of patients present with symptoms related to mitral valve obstruction, systemic embolization, or constitutional symptoms [1,2]. Neurologic symptoms, even without concomitant cardiac symptoms, may be the presenting sign and are most commonly due to cerebral ischemic events from the embolization of either tumor fragments or thrombi formed on the tumor surface [1-3].

Echocardiography is commonly utilized for the initial diagnosis and characterization of cardiac tumors, with transesophageal echocardiography (TEE) being preferred over transthoracic echocardiography (TTE) [4]. After an initial diagnosis, cardiac magnetic resonance imaging (MRI) can be used for better characterization and localization of cardiac masses to guide management, including differentiating between myxomas and thrombi [5]. The treatment for atrial myxoma is surgical removal, and the recurrence rate is favorably low [1,2,6].

We report the case of a 42-year-old male with a left atrial myxoma presenting as an ischemic stroke in the setting of a recent COVID-19 infection. The case demonstrates the importance of evaluating young patients presenting with multifocal stroke for cardiac tumors to allow prompt resection and prevention of further embolic events.

This article was previously presented as a poster at the 2021 American College of Physicians Michigan Chapter Annual Scientific Meeting on October 14, 2021.

## Case presentation

A 42-year-old male with a past medical history of uncontrolled type 2 diabetes mellitus and hypertension presented to the emergency department approximately eight hours after he experienced acute onset numbness, heaviness, and weakness in his left arm along with left leg weakness. He first noticed the symptoms earlier in the day; however, they persisted and progressively worsened for several hours, resulting in a fall at home in which he could not use his arm to catch himself. He reported no preceding pre-syncopal symptoms or aura but reported hitting his head on a hard surface. His review of systems was significant for an unintentional 30-pound weight loss over the previous six months, but otherwise, he denied headache, syncope, seizure-like activity, urinary or bowel incontinence, and tongue biting. Notably, he denied taking his antihypertensive and diabetic medications, including lisinopril, metformin, liraglutide, and pioglitazone, for over a year. Other notable history included a COVID-19 infection two months prior to symptom onset that did not require hospitalization.

On presentation to the emergency department, the patient was hypertensive to 176/110 mmHg, with all other vital signs within normal limits. Physical examination revealed mild ecchymosis on the right side of the head. Cranial nerves II-XII were intact with no facial droop, there was left-sided pronator drift with 3/5 strength in the proximal left upper extremity and 2/5 strength in the left elbow flexion and extension. Left-hand grip was significantly weakened, and there was decreased sensation of the left upper extremity to fine and dull stimuli. The left lower extremity strength was 4/5. Sensation and strength of all other extremities, as well as cerebellar testing, were normal. His reflexes were 2+ and symmetric throughout.

**Figure 1:**
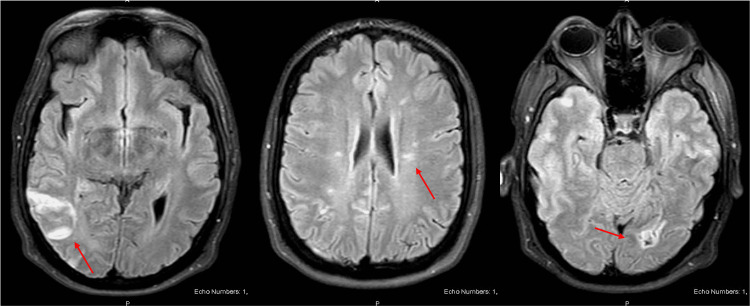
Non-contrast brain MRI images showing evidence of strokes (red arrow) in the right middle cerebral artery territory (left image), left cerebral hemisphere (middle image), and left posterior cerebral artery territory (right image). MRI: magnetic resonance imaging

**Figure 2:**
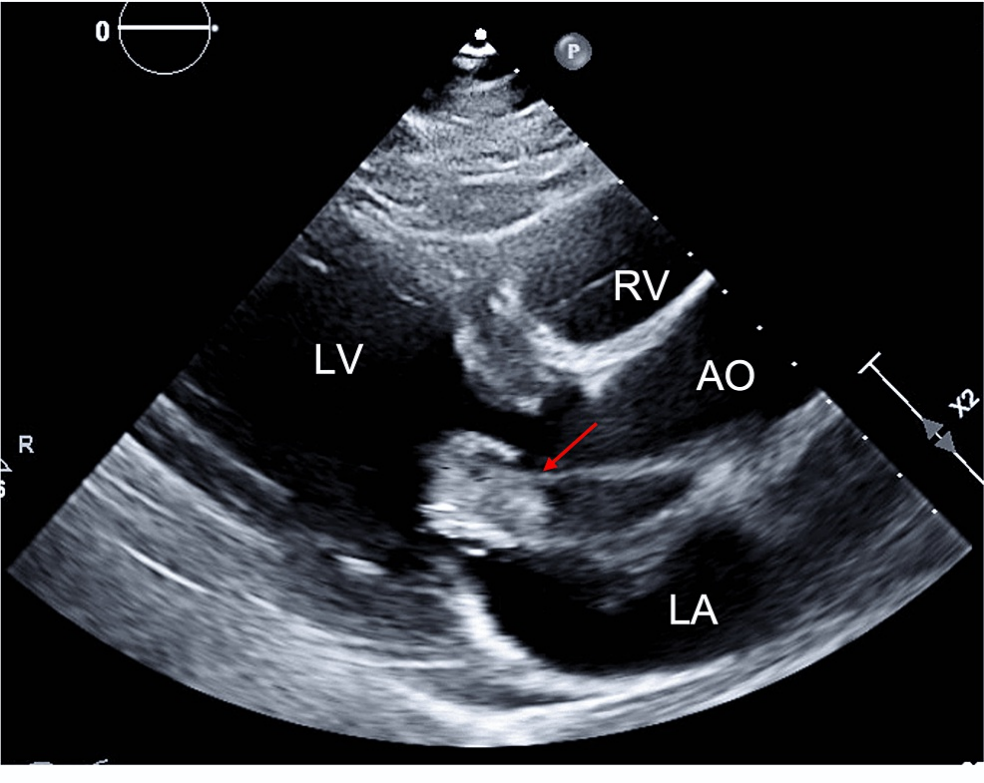
TTE with parasternal long-axis view demonstrating a 4.65 × 2 cm mass (red arrow) in the left atrium attached to the atrial septum. TTE: transthoracic echocardiogram

**Figure 3:**
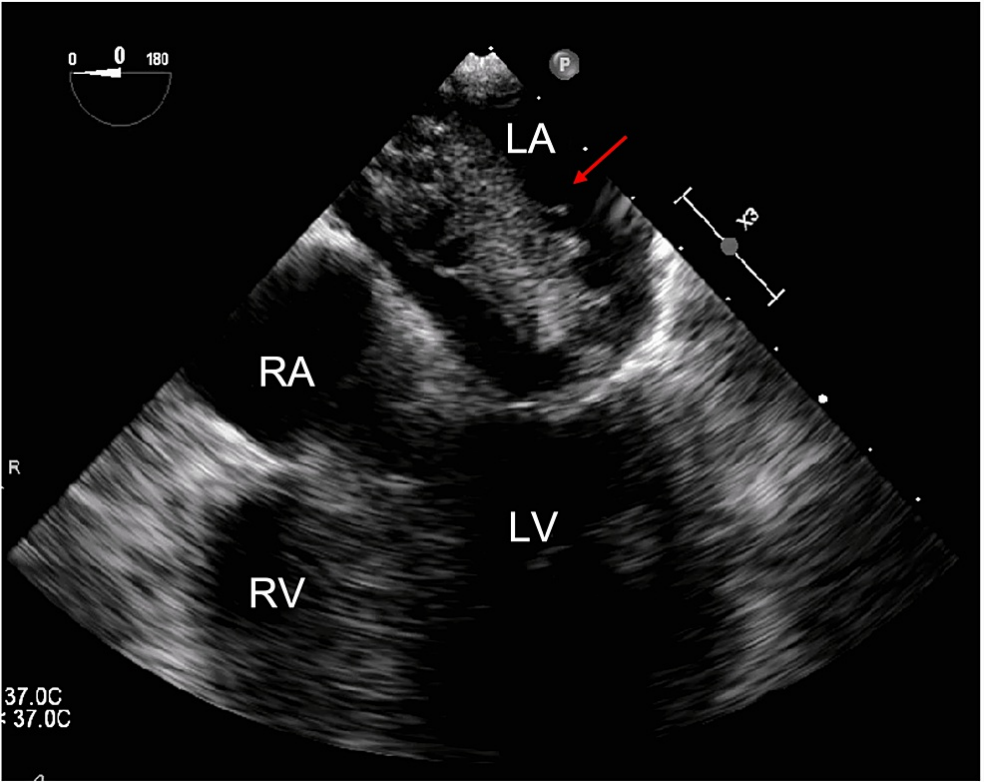
TEE with four-chamber view illustrating a left atrial mass (red arrow) measuring 5 × 2 cm attached to the atrial septum prolapsing the mitral valve. TEE: transesophageal echocardiogram

The initial blood work was significant only for hyperglycemia to 310 mg/dL and a hemoglobin A1C of 12%. An electrocardiogram (ECG) illustrated normal sinus rhythm. A computed tomography (CT) of the head without contrast revealed evidence of an old left posterior cerebral artery infarct but no acute process. A stroke workup was initiated, and a brain MRI without contrast was significant for an acute infarct in the right middle cerebral artery/watershed territory with microhemorrhages, foci of acute versus subacute infarcts in the watershed distribution of the left cerebral hemisphere, and signs of an old hemorrhagic infarct in the territory of the left posterior cerebral artery (Figure 1). Carotid duplex ultrasound showed no significant arterial stenosis, and TTE revealed normal left ventricular function with an estimated ejection fraction (EF) of 65%, mild dilation of the left atrium, and mobile left atrial septal mass measuring 4.65 x 2.0 cm (Figure 2). Subsequent TEE showed a left atrial mass attached to the atrial septum measuring 5 × 2 cm and prolapsing the mitral valve, suspicious of atrial myxoma (Figure 3). Left heart catheterization with coronary angiography was recommended by the cardiothoracic surgery team to identify the presence of clinically significant coronary artery disease that could be corrected at the time of sternotomy and myxoma removal if necessary. The procedure was performed and was significant only for 50%-60% stenosis of the proximal left anterior descending artery.

The patient did not receive thrombolytic therapy as his last known normal was over 4.5 hours prior to presentation. Given the findings of multifocal strokes and the location of his acute stroke, he was not a candidate for mechanical thrombectomy. He was started on an unfractionated heparin continuous infusion after discussions with the patient’s consulting neurologist and cardiothoracic surgeon. To optimize his chronic medical conditions, the patient was restarted on his previous medications including atorvastatin, lisinopril, and metformin, with additional basal insulin for glycemic control. Due to the patient’s hemoglobin A1C of 12%, the risk of sternal infection following sternotomy for myxoma removal was considered too high. Weighed against the risks of having another embolic event while awaiting optimization of glycemic control, the decision was made to transfer the patient to an advanced facility capable of immediate minimally invasive robotic myxoma excision. The patient underwent successful robotic removal of left atrial myxoma, complicated only by postoperative fluid overload that resolved with pharmacologic diuresis. The pathology results illustrated atrial myxoma with an associated fibrin thrombus. He was discharged on aspirin and atorvastatin for secondary stroke prevention as well as his home hypertension and diabetes medications with instructions to follow up with both his primary provider and neurology with emphasis on control of blood pressure and blood glucose.

The patient reported no new concerns or neurologic symptoms at a subsequent two-month follow-up with his cardiothoracic surgeon and neurologist, who continued the patient on aspirin and atorvastatin for stroke prevention as well as his antihypertensive and diabetic medications.

## Discussion

Atrial myxomas make up approximately 80% of primary heart tumors [7]. Neurologic symptoms may be the presenting sign in up to 12% of patients, most commonly due to cerebral infarctions and less commonly as hemorrhagic strokes due to aneurysm formation, which may occur after tumor excision [3,7,8]. Our case is consistent with these findings as our patient initially presented with ischemic stroke, although it is unclear if the 30-pound weight loss he experienced prior to presentation was a constitutional symptom of his myxoma or due to his uncontrolled diabetes mellitus.

A recent case review series of 130 patients with left atrial myxomas found that 22 patients initially presented with neurologic symptoms, and cerebral vascular events most commonly involved the middle cerebral artery [9], as was the case with our patient. The authors also reported that irregularity of the myxoma surface correlates to a greater risk of embolism by contributing to fragmentation and subsequent embolism of tumor fragments [10]. While we cannot comment on the surface irregularity of our patient’s tumor, pathology results showed attached fibrin and thrombus, and it has been shown atrial myxomas that cause embolic phenomena are more likely to have a surface thrombus [10].

There are no clear guidelines for the use of anticoagulation in the setting of atrial myxoma. One study found that even with antiplatelet or anticoagulation therapy, embolic phenomena occurred in 46% of patients with cardiac myxomas [11]. However, there are cases that document resolution of symptoms and favorable outcomes using anticoagulation, such as unfractionated heparin, which was used in our patient, prior to myxoma removal [12]. Given the mixed results and lack of evidence-based guidelines, it appears reasonable to weigh the risks and benefits of starting systemic anticoagulation on a case-by-case basis for patients with suspected thromboembolic phenomena caused by atrial myxoma. In our case, in discussion with the patient’s multiple consultants, it was decided that the potential benefit of lowering the risk of further embolism and preventing further disabling neurologic symptoms, especially given our patient’s history of multiple cerebral events of differing ages, outweighed the increased risk of hemorrhage even in the setting of intracranial microhemorrhage.

In a young patient with ischemic stroke, considering other thrombotic risk factors is crucial, and COVID-19 should be considered in the appropriate setting. Infection with COVID-19 has been associated with increased incidences of both venous and arterial thrombosis, although it appears that the risk of arterial thrombosis is much lower [13,14]. One case series from a New York City health system describes five patients under the age of 50 who experienced large cerebral vessel ischemic occlusions, a sevenfold increase from the previous baseline stroke rate in such a population [15]. Other case series have also reported on patients with COVID-19 infection presenting with ischemic stroke at lower but still concerning rates [13,16]. The risk of arterial thrombosis in COVID-19 patients most commonly occurs in critically ill patients, male patients, and those with comorbid conditions [17]. Further investigations are underway to better understand the mechanism of the hypercoagulable state seen in COVID-19 patients, but endothelial injury, complement activation, platelet activation, and pro-inflammatory cytokine release have been implicated [14,17,18].

While our patient only had a mild COVID-19 infection that did not require hospitalization, even young non-critically ill COVID-19 patients are at risk of thrombotic events and stroke [15,19]. The hypercoagulable state and risk of stroke may persist even after the initial infection as part of post-acute COVID-19 syndrome [18]. While the duration of thromboembolic risk secondary to COVID-19 is yet to be determined, some experts suggest a six-week course of anticoagulation [18]. Our patient’s infection occurred two months prior to his stroke, and it is unclear whether the infection played a role in making him hypercoagulable, thereby contributing to thromboembolization from his myxoma. It also is unclear if infection with COVID-19 contributed to his non-acute cerebral vascular injuries seen on imaging.

## Conclusions

Our case demonstrates the importance of considering atrial myxoma and other cardiac tumors when evaluating suspected stroke in a young patient. Furthermore, even if a patient has cardiovascular risk factors or a current or a recent COVID-19 infection, a thorough workup for a cardiac source of embolism should still be conducted. Prompt diagnosis of left atrial myxoma can lead to expedited tumor removal and prevention of further embolism and stroke, which may cause disabling symptoms and death in some cases.
